# Effect of Low-Thermal Treatment on the Particle Size Distribution in Wood Dust after Milling

**DOI:** 10.3390/polym15041059

**Published:** 2023-02-20

**Authors:** Martin Júda, Maciej Sydor, Tomasz Rogoziński, Martin Kučerka, Marta Pędzik, Richard Kminiak

**Affiliations:** 1 Department of Woodworking, Faculty of Wood Science and Technology, Technical University in Zvolen, 960 01 Zvolen, Slovakia; 2 Department of Woodworking Machines and Fundamentals of Machine Design, Faculty of Forestry and Wood Technology, Poznań University of Life Sciences, 60-637 Poznań, Poland; 3 Department of Furniture Design, Faculty of Forestry and Wood Technology, Poznań University of Life Sciences, 60-637 Poznań, Poland; 4 Faculty of Natural Sciences, Matej Bel University, 974 01 Banská Bystrica, Slovakia; 5 Center of Wood Technology, Łukasiewicz Research Network—Poznań Institute of Technology, 60-654 Poznań, Poland

**Keywords:** thermal modification, woodworking, birch wood, beech wood, alder wood

## Abstract

The thermal treatment of wood can improve the appearance of the wood product’s surface, its dimensional stability, and resistance to fungal attacks. However, the heat treatment changes the technological properties of wood, making it a new engineering material. This work investigates the effect of the low-thermal treatment of birch wood (*Betula pendula* Roth.), European beech wood (*Fagus sylvatica* L.), and alder wood (*Alnus glutinosa* L.) on the fine dust particles creation during woodworking. The samples of thermally treated wood with temperatures commonly used for the change of wood colour (105, 125, and 135 °C) were compared with reference samples made of natural wood. All 12 variants of the tested woods were milled using the 5-axis CNC machining center (20 mm diamond cutter, rotational speed 18,000 rev·min^−1^, the depth of cut 3 mm, feed rates of 2, 4 and 6 m∙min^−1^). A sieving analysis method allowed measuring the dust particle size distributions in all dust samples. The experiment’s result analysis points out that wood type, thermal treatment, and feed rate meaningfully affect the size distribution of dust particles. Compared to birch wood and beech wood, the milling of alder wood samples created a much higher content of the finest dust particles, with particle sizes smaller than 0.032 mm. Increased temperatures in thermal treatment increase the share of fine dust particles with sizes smaller than 0.125 mm, compared to wood in its natural state. Milling with a lower feed rate (2 m·min^−1^) creates finer dust than processing with higher feed rates (4 and 6 m·min^−1^). Generally, the milling of alder in a natural or thermally treated state is a source of fine dust particles, particularly at low feed speed-rate milling, compared to birch and beech wood. In general, these results indicate that the low temperature thermal treatment parameters attribute new technological properties to all thermally modified types of wood tested.

## 1. Introduction

The wood processing industries are sources of dust emissions [1]. Efficient dedusting of wood material-removal processes is recommended because wood dust poses: a health risk to workers [2,3,4], an explosion risk [5], deteriorates the manufacturing technology [6], and can be used as a by-product [7,8]. Exposure to wood dust causes occupational diseases such as skin and mucous membrane irritation, various allergies, and chronic respiratory diseases [9,10,11]. Studies also prove that geographic region (dominant in hardwood or softwood species) has a significant impact on the relationship between wood dust and lung cancer [12]. Unfortunately, cumulative exposure to dust from softwood in particular is associated with an increased risk of upper respiratory tract cancers e.g., nasal adenocarcinoma [13,14].

Small particles of wood dust become airborne; however, the human upper respiratory system can filter out the larger particles, but the smallest particles penetrate deep into the lungs causing damage and scarring to the lung tissue. According to Lim et al. 2012 [15], worldwide particulate matter emissions are the main environmental issue of health illnesses, and air pollution causes premature deaths. Since 1995, the International Agency for Research on Cancer (IARC) has classified wood dust as a carcinogen of the human (group I) that induces nasal, pharynx, and larynx malignancies [16,17]. European Union directives identify hardwood dust as a human carcinogen and from January 2023 establish an occupational exposure limit (OEL) of 2 mg∙m^−3^ of inhaled hardwood particles over an 8-h reference period. [16]. There is a difference between “inhalable” and “respirable” wood dust. “Inhalable” refers to particles that can be taken into the lungs. According to technical standards, inhalable wood dust is composed of particles with an aerodynamic diameter smaller than 100 μm [18,19]. On the other hand, “respirable” refers to particles that are small enough to penetrate deep into the lungs and potentially cause harm to human health. Respirable particles are generally smaller than inhalable particles and have sizes of less than 10 μm.

After dispersing in the air, the wood dust fraction in the size range of 10 and 100 μm settle in the skin and eyes. They penetrate the human respiratory system, causing benign pathologies [20]. Furthermore, particles smaller than 10 μm can reach the bronchi, while particles smaller than 2.5 μm can get into the pulmonary alveoli, causing respiratory diseases [21]. The result of the adverse influence of industrial dust depends on the type of inhaled dust, the structure of the respiratory tract, and the process of breathing [22]. Dust created during woodworking presents one of the most significant practical problems in the working environment [23].

Wood dust consists of small wood chips. The wood chips’ size depends mainly on the machining process, including the state of the tool cutting edges and the cutting process’s technical conditions and technological parameters [24,25,26]. The finest particles’ content also depends on the processed wood material [27]. The dust in the atmosphere is always polydisperse and contains particles of various dimensions [28]. The dustiness resulting from the mechanical processes is an essential issue of working safety in the wood processing industries.

Thermal treatment (thermal modification, hydrothermal treatment) processes are applied to a well-established commercial technology for improving wood’s appearance, dimensional stability, and durability. There are reports about hygrothermal treated hardwood [29,30,31], softwood [32,33], and bamboo [34]. Heat-treated wood, due to its properties, is suitable for flooring, various building facade elements, and especially for humid areas such as saunas and bathrooms. Thermal treatment can be a way to improve wooden products’ dimensional stability and resistance against fungal attacks. However, there are also undesired side effects caused by high temperatures, around 150 °C, such as deteriorated resistance to shock, lowered modulus of elasticity (MOE), bending strength (MOR), compressive resistance, shear strength, and abrasion resistance [35]. Worn physical properties make the wood more fragile, which could limit the range of usability of heat treatment technologies for wood products [36,37,38]. The loss of hemicelluloses during thermal treatment is one of the explanations for the decrease in strength properties and increase in dimensional stability [35]. Chemical methods of wood modification due to adverse environmental effects are less preferred and in this regard heat treatment is recommended, as it is more environmentally friendly. [39,40,41]. Significant changes in wood properties affect dust formation during wood processing, and the thermal treatment changes the particle size distributions of the dust fractions [42]. Technological innovations in wood modifications and their mechanical processing can be a potential source of increased dustiness in working zones. Based on dust creation, understanding how much technological conditions of milling thermally treated wood species are responsible for this phenomenon can be helpful. However, it is not easy to determine whether the high content of fine particles is due solely to the heterogeneous structure of materials, cutting tool geometry and CNC machine conditioning, etc. This justifies the study of the properties of the dust emitted during wood processing.

The study aims to verify how, in the different wood species, the modification temperature and feed rate change the wood dust parameters, making it dangerous to health.

## 2. Material and Methods

### 2.1. Tested Wood Species

The experimental study included 180 samples of three kinds of wood: birch (*Betula pendula* Roth), European beech (*Fagus sylvatica* L.), and alder (*Alnus glutinosa* L.) originating from Slovakia). The elements designed to generate dust were prepared as blocks, with width *w* = 80 mm, length *l* = 600 mm, and thickness *t* = 40 mm, with an initial relative moisture content in the range of 54.7–58.2%. The moisture content of samples was measured by laboratory gravimetric method in accordance with standards EN 13183-1 [43] using a laboratory kiln MEMMERT UM110m, drying to constant non-changeable weight at temperature t = 103 ± 2 °C. From the difference in weights before drying (m_w_) and after drying (m_0_) we calculated the moisture content by Equation (1):

(1) $$w=\frac{m_{w}-m_{0}}{m_{0}}\cdot 100\; [\%]$$

The moisture content of dried samples at moisture *w* = 12 ± 0.5% was performed by an electrical hygrometer FMD6, which gives a value of moisture in the depth of 2–3 mm below the surface of a sample.

For density measurements, the methodology was the same as the in previous case. The samples were first dried in a laboratory kiln MEMMERT UM110m, to constant non-changeable weight at temperature *t* = 103 ± 2 °C. After the samples dried out, they were placed in a glass exicator. After their cooling down to the temperature of the environment, density was measured on a digital density meter KIT 128 by RADWAG according to standards STN 49 0108 and internal methodology for measurement of density lower than φ ≤ 1000 kg·m^−3^ at Technical University in Zvolen. The methodology is based on Archimedes’ law, where the mass of the sample without moisture (m_0_) is weighted on air and in distilled water. The volume of the sample is measured from the mass balance of the samples from Equations (2) and (3):

(2) $$m_{0}-m_{0}{}^{*}=V\cdot \rho_{H20}\cdot g\; [\mathrm{kg}]$$

(3) $$V=\frac{m_{0}-m_{0}{}^{*}}{\rho_{H20}\cdot g}\; [m^{3}]$$

The density of the sample is calculated from Equation (4):

(4) $$\rho_{0}=\frac{m_{0}}{V}=\left(\frac{m_{0}}{m_{0}-m_{0}{}^{*}}\right)\cdot \left(\rho_{H20}\cdot g\right)\; [\mathrm{kg}\cdot m^{3}]$$

The wood types tested were divided into four groups, each consisting of 45 samples. The control group remains unmodified (denoted as N). The second group was heat-treated with steaming mode I (T1), the third group was heat-treated with steaming mode II (T2), and the samples in the fourth group were heat-treated with the III steaming mode (T3). Table 1 summarizes the tested wood samples’ physical properties and thermal treatment variants.

### 2.2. The Thermal Treatment

**Figure 1:**
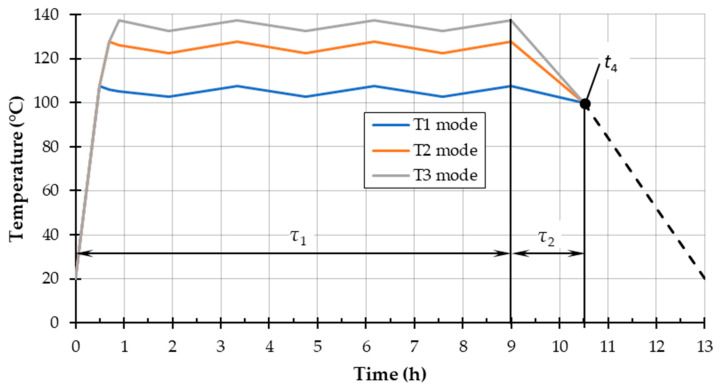
Temperature diagram of the heat treatment process in the pressure by steam used in an autoclave (adapted from [44].)

The thermal treatment was carried out in an industrial kiln by the company Sundermann s.r.o., Slovakia, in the pressure autoclave (type APDZ 240, Himmasch AD, Haskovo, Bulgaria) under the action of saturated water vapor. Figure 1 shows the technological process diagram for the thermal treatment with saturated steam.

Temperatures *t*_min_ and *t*_max_ are intervals in between saturated water vapor supplied to the autoclave during the thermal treatment process. The temperature *t*_4_ is the saturated steam pressure parameter at the end of the described process. Table 2 summarises the parameters of the used steaming modes.

Finally, all studied materials—non-thermally treated and thermally treated, were dried at a low temperature, without affecting the colour change of the wood, to a moisture content of *w* = 12 ± 0.5% in a conventional hot air dryer (type SUZAR KC 1/50, SUZAR s.r.o., Považany, Slovakia) [44].

### 2.3. Creating Dust by Milling

The milling was carried out using a 5-axis CNC machining center (Tech Z5, SCM Group, Rimini, Italy). This CNC machine is commonly used in the wood industry. It is equipped with a vertical spindle, an electric motor of 7.5 kW, and an adjustable rotational speed ranging from 600 to 24,000 rev·min^−1^. The CNC machine consists of 6 adjustable beam supports equipped with vacuum clamps to mount the machined workpiece. The machining was carried out with the chip extraction installation turned off due to a decision to use custom extraction equipment to exceed the extraction mechanism’s effectiveness.

**Figure 2:**
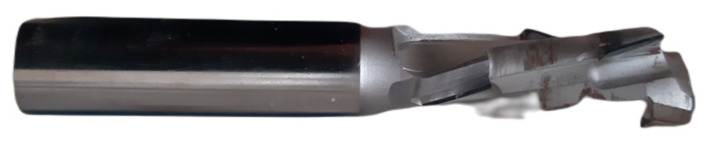
A negative shank milling cutter with two diamond cutting blades (Source: IGM Tools and machines).

The tool used was a new negative shank milling cutter with two diamond cutting blades, 20 mm diameter, and 26 mm effective cutting length (IGM tools and machines, Tuchoměřice, Czech Republic) commonly used in roughing operations (Figure 2). This tool has been chosen for its common use. Only one tool was used during the experiment due to the manufacturer’s expected tool lifetime, which would not require a new cutting tool. No blunting or different tool wear forms were measured before and after the experiment.

The experimental workpieces were milled at a rotational speed of 18,000 revs·min^−1^ and feed rates of 2, 4, and 6 m·min^−1^. The direction of the milling tool to the workpiece was down-milling (climb milling). The depth of cut was 3 mm, and this value remained constant during the experimental machining process. The cutting parameters used in the experimental study were based on the actual technological processes in which those materials are usually cut in the roughing process. Materials were milled until at least 50 ± 1 g of sawdust samples were gathered. After the milling, the entire area for collecting samples was cleaned out before the next sample was machined.

### 2.4. Dust Particles Size Distribution

The particles created during the milling were collected by a filtration textile (Main Filter HEPA-HF-CT26/36/48 PTFE) with filtration of 99.97% of particles down to 0.3 μm in size. The particle size distribution (PSD) was measured by sieving analysis with a set of stacked sieves. The set was placed at the vibration stand (AS 200c, Retsch GmbH, Haan, Germany). Sieving parameters following standards of ISO 3310-1 were used (sieve interruption frequency *f*—20 s, amplitude—2 mm∙g^−1^, screening time *t* = 15 min, and the weight of the samples *w* = 50 g). The set of sieves consists of sieves with sizes in the interval of 2, 1, 0.5, 0.25, 0.125, 0.063, and 0.032 mm, and the bottom collector (collecting the finest particles with sizes below <0.032 mm). All fractions of particles separated in sieve analysis were weighted with a laboratory balance (510/C/2, Radwag Balances and Scales, Radom, Poland) with an accuracy of 0.001 g. The proportions of the particular sieve fractions were calculated based on the weight of the fractions.

Three measurements were taken for every combination of used material, thermal treatment mode, and feed rate (birch-beech-alder, N-T1-T2-T3, and vf2-vf4-vf6). Granulometric analysis was performed on 108 samples in total. Samples were analyzed by the sieve analysis off-site in a laboratory condition by sieve analysis. The following symbols mean, for example:

- Birch-N-2—birch wood in a natural state (not thermally modified) and milled at a feed of 2 m·min^−1^
- Beech-T1-4—beech wood thermally treated in T1 mode and milled at a feed of 4 m·min^−1^

### 2.5. Statistical Analysis

Statistical analysis was carried out for each combination of granular particle size fraction range (i.e., coarse, medium coarse, and fine fraction) using the statistical software Statistica 12 (Tibco, Palo Alto, CA, USA). A multiple factors ANOVA was used for 0.125, 0.063, 0.032, and for <0.032 mm particle fractions. Tested factors were material, thermal treatment, feed rate, and combinations of those factors: material—thermal treatment, material—feed rate, and material—thermal treatment—feed rate.

## 3. Results and Discussion

The average density of natural birch wood was 649 kg/m^3^. As a result of thermal modification, it dropped initially by 3.7%, and at maximum temperature 135°C by 4.3% to 621 kg/m^3^. A similar decrease in density and a gradual relationship was observed for alder wood. From 523 kg/m^3^ for natural wood, it went sequentially to 510 kg/m^3^ (a decrease of 2.5% due to modification at 125 °C), to 500 kg/m^3^ a decrease of 4.4% in density at 135 °C. It can be seen that as the temperature increased, the density of these species decreased.

Beech wood behaved differently during thermal modification. The initial density was 683 kg/m^3^, followed by a decrease in value to 670 kg/m^3^ and an increase to 684 kg/m^3^. In the Kvietkova et al. 2015 [45] work, they observed a 5% decrease in beech density during thermal modification at 190 °C. Beech wood was modified at 130 °C, obtaining a decrease in density of about 2.3%, similar to our work. This result was attributed to the properties of the wood raw material and such a small increase in density was considered insignificant [36,46]. The reduction in wood density is probably due to a decrease in wood weight due to loss of water as well as the release of volatile by-products. In addition, a further increase in temperature to almost 140 °C may have already contributed to the loss of wood mass through surface changes in the structure of polymeric cell wall components, mainly carbohydrate components.

**Figure 3:**
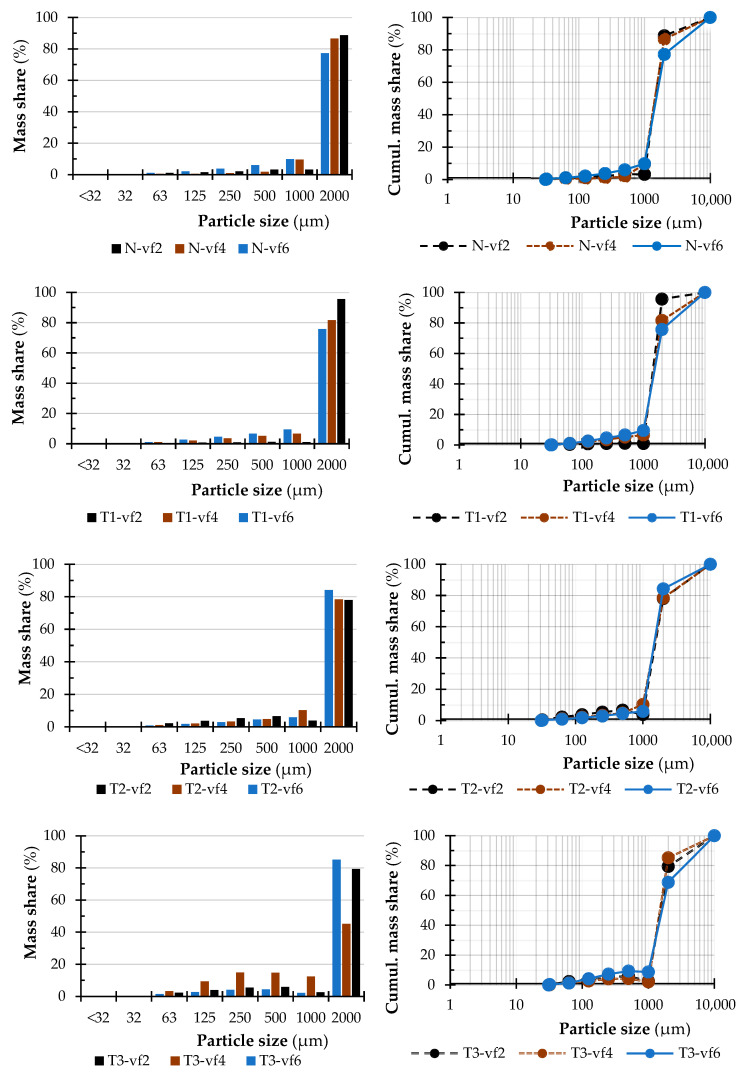
Particle size distribution (PSD) of birch dust in dependence on thermal treatment and feed rate.

**Figure 4:**
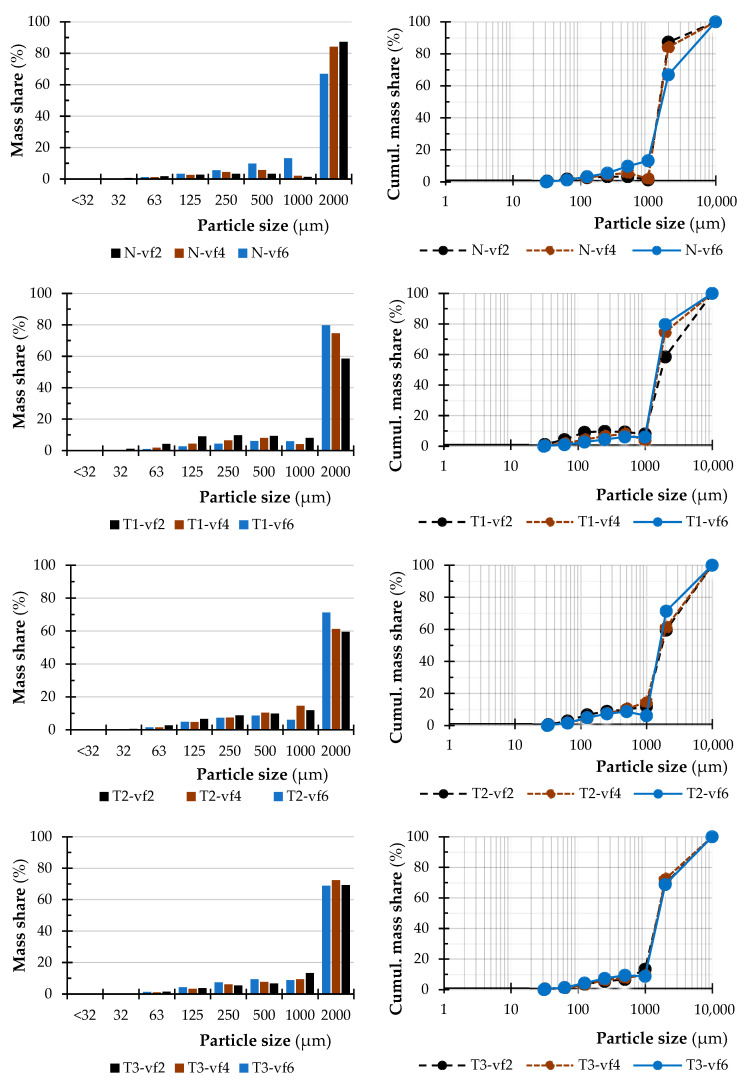
Particle size distribution (PSD) of beech dust in dependence on thermal treatment and feed rate.

**Figure 5:**
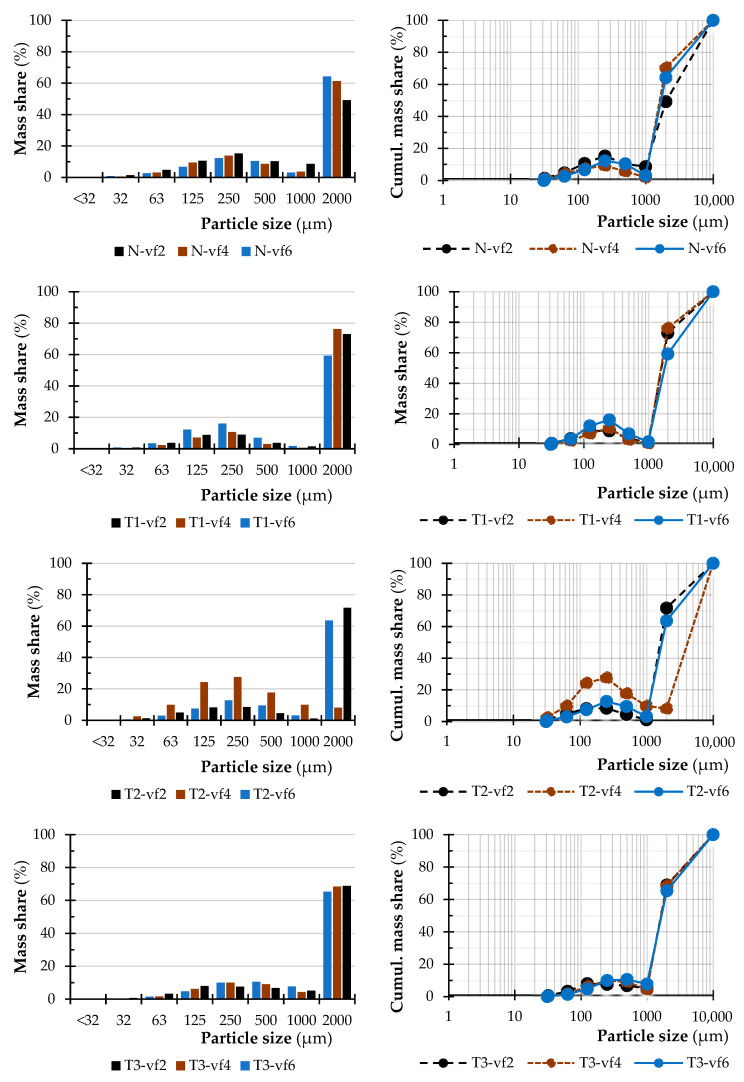
Particle size distribution (PSD) of alder dust in dependence on thermal treatment and feed rate.

The particle size distributions of birch, beech, and alder dust were measured by sieve method. Figure 3, Figure 4 and Figure 5 summarise the results of the measurement. It is important to note that no wood dust particles were observed in the range of 0.032 to <0.032 mm during the experimental process. Some small fractions were observed during the weighting analysis, but those values remained almost negligible overall.

This overall interpretation of results provides preliminary and general information about the wood dust particles of natural and thermally treated wood specimens formed during milling at various feed rates, especially using a CNC milling machine. Due to limited space, the type of materials, the mode of thermal treatment, and the feed speeds are in the text represented by short format names.

Most of the dust particles generated during the milling of birch-, beech-, and alder wood are in the range of the so-called coarse fraction (particles size in the range of 1.0–2.0 mm). This range represents 96% to 66% of the total dust particles. In the case of birch wood, it ranges mostly from 97% to 81%; in the case of beech wood, from 88% to 71% and in the case of alder wood, from 76% to 57% of the total mass of the analyzed sample. Moreover, the results of Kminiak et al. 2021 showed that the processing of natural wood species such as beech, oak, and spruce is mainly characterized by the formation of coarse dust fractions (1.0–2.0 mm sieves) [47]. It was observed that sample birch-T3-4 has a significant difference in PSD compared to the rest of the presented samples in that range of tested material. The same can be seen in the case of sample beech-T1-2 and alder-T2-4 which also has a significant difference in PSD to the rest of the presented samples in that range of tested material.

A comparison of the particle sizes of wood dust generated during milling using three feed speeds indicates, at least to some extent, that milling at a lower feed speed results in the creation of smaller dust particles.

In its natural state, birch wood, milled at a feed of 2 m·min^−1^ generates around 2.86% of the total mass of fractions in the range of the so-called fine fractions. Milling this material at a feed speed of 4 m·min^−1^ lowered the total amount of this fine fraction to 1.18%. Further increasing the feed speed to 6 m·min^−1^ increased the range of fine fraction to 3.38%. Compared to beech wood material, the content of the fine fraction milled at a feed speed of 2 m·min^−1^ is higher. In this case, generated dust fractions from the process in the range of fine fractions represent around 4.9% of fine fractions. At a feed of 4 m·min^−1^ the fine fraction content is again lowered to around 3.94%, and further increased the feed to 6 m·min^−1^ results, as in the previous case, in increasing fine fractions to 4.69% and not lowering it. In the case of alder wood, the content of fine fraction milled at a feed of 2 m·min^−1^ generated from the milling process is around 16.67%. It is much higher than compared to previous materials, birch wood, and beech wood. Increasing the feed speed to 4 m·min^−1^ lowered the volume number of fine fractions to approximately 12.84% and to approximately 10.06% at a maximum feed speed of 6 m·min^−1^.

The feed speed affects the amount of dust produced by milling normal alder wood. Increasing the feed speed has a beneficial effect on reducing the amount of dust formed with the most dangerous grain size. A reduction in the fine fractions to about 10.06% can be observed, which did not increase at the maximum speed as in previous cases of the tested materials. The relationship between increasing feed speed and reducing the amount of fine dust formed is not confirmed for birch and beech. Similarly, in the work of Kminiak et al. 2021, no apparent effect of feed rates of 1, 3 and 5 m·min^−1^ on the formation of fine dust of beech wood was determined, obtaining values of 3.46, 5.41, and 3.67%, respectively [47].

**Figure 6:**
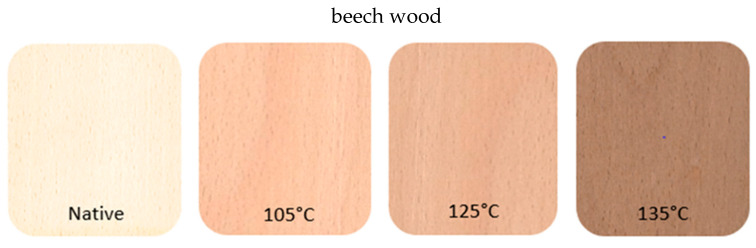

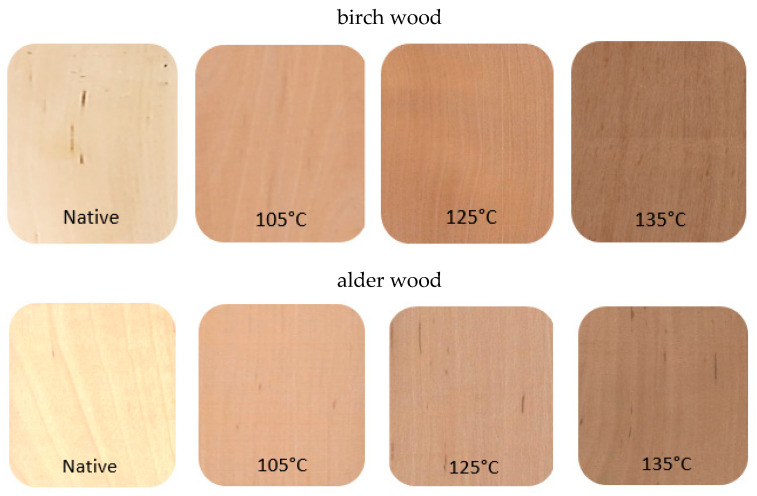
Surface of samples before and after thermal modification.

Figure 6 shows the surfaces of the samples before and after thermal modification at 105, 125 and 135 °C.

Thermal modification affected the color of the wood. The higher the modification temperature, the darker the wood became. Even at the lowest modification temperature, the difference in the color of the wood surface relative to the natural sample is significant. The ability to change the color is an undoubted advantage of this method and affects the aesthetic qualities. No undesirable color changes, such as sinkages, were observed on the samples.

Thermal treatment T1 mode (105 °C) did not significantly affect the total amount of fine dust formed for birch and alder relative to natural wood (for birch a decrease of 0.74%, for alder an increase of 0.55%), taking into account the sum of the amount of dust from all feed rates. However, for beech wood, the modification resulted in a significant increase in the amount of dust from 13.5% for wood before modification to as much as 25.01% after modification. For birch wood modification T1 in combination with a feed speed of 2 m·min^−1^ created around 1.28% of fine dust particles in this range, which is the most favourable option regarding feed speeds. Beech wood created around 14.52% of fine fractions, and alder wood created around 13.15% of fine dust particles. With a combination of feed speed of 4 m·min^−1^ in birch wood around 3.18% of fine dust particles were created. In beech wood’s case around 6.63%, and in alder wood around 9.68% of fine dust particles were created, and the last combination with a feed speed of 6 m·min^−1^ in the case of birch wood 3.70% was created, in beech wood around 3.88%, and in alder wood’s case around 16.22% of fine dust particles were created in this combination of thermal treatment mode and feed speed.

Dust particle size analyses of beech wood studied by Rogoziński et al. 2021 [26] showed that sawing beech wood dried with a mixture of warm air and steam at 105 °C results in an increase in the content of fine dust particles compared to air-dried wood, especially at low feed rate. Admittedly, this is not thermally treated but dried wood; however, the cited previous study points out a similar relationship to the results described in this work regarding wood species and feed speed [26].

In the T2 thermal treatment mode (125 °C) with a combination of feed speed of 2 m·min^−1^, a significant increase for birch wood to approximately 6.25% of dust particles in the dust fraction range was observed for the beech wood it was around 9.89%, and for the alder wood around 14.31% with a combination of feed speed of 4 m·min^−1^ dust particles created around 3.39% for birch wood, 6.44% for beech wood. Surprisingly, as much as 36.74% was fine fraction dust of alder wood, making the combination of T2 modification and a feed speed of 4 m∙min^−1^ by far the least favourable variant of processing generating such a high amount of the most harmful particles. For comparison, with the combination of this thermal modification mode and feed speed of 6 m·min^−1^ in birch wood, around 2.72% of fine dust particles were created. Beech wood created around 6.74%, and alder wood around 11.03% of fine dust particles.

However, for the thermal treatment mode T3 (135 °C) of birch wood and beech wood, an increase in dust formation was observed for each feed speed variant. For a feed speed by 2 m·min^−1^, it was observed. That birch wood formed around 6.62% of fine dust particles (a slight increase with respect to T2) is created, beech wood around 5.41% and alder wood’s case around 11.77% of fine dust particles. With the combination of feed speed of 4 m·min^−1^ for the birch wood case, 12.84% of fine dust particles are created, making this the most unfavourable option for this species. For beech wood around 4.48%, and for alder wood case around 8.21% of fine dust particles were created. In the last combination with the feed speed of 6 m·min^−1^, birch wood created around 4.25% of fine dust particles, beech wood around 5.84%, and alder wood around 6.46%. An increase in the modification temperature to a maximum of 135 °C (T3) for alder wood contributed to a reduction in the amount of fine dust fraction formed, as did an increase in the feed speed to a maximum of 6 m∙min^−1^. It was for this combination that, by far, the most favourable processing variant was obtained. The percentage differences obtained between the variants are often not large, so their significance cannot be an explicit confirmation of impact, although some differences are apparent. These differences may also be due to each wood sample’s structure and specific properties. The study by Kučerka and Očkajová 2018 [48] presents the grain composition of abrasive dust of spruce and oak wood subjected to thermo-modification at 160 °C, 180 °C, 200 °C and 220 °C. They found that the percentage of particles below 0.80 mm of wood treated at 160 °C, 180 °C, and 200 °C was similar for natural wood. They observed a smaller proportion of the fine dust fraction at 220 °C in all samples tested, resulting in a reduction in wood density. Their results obtained for samples of other species and modified at much higher temperatures confirm our observations [48].

**Figure 7:**
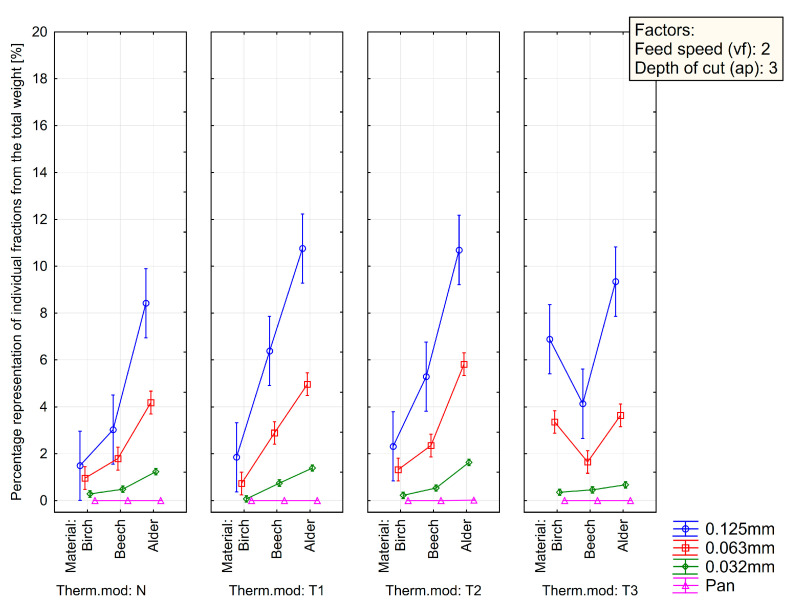
Sieve analysis of fine fraction obtained by the depth of cut by 3 mm and the feed rate of 2 m·min^−1^ (vf2- ap3).

**Figure 8:**
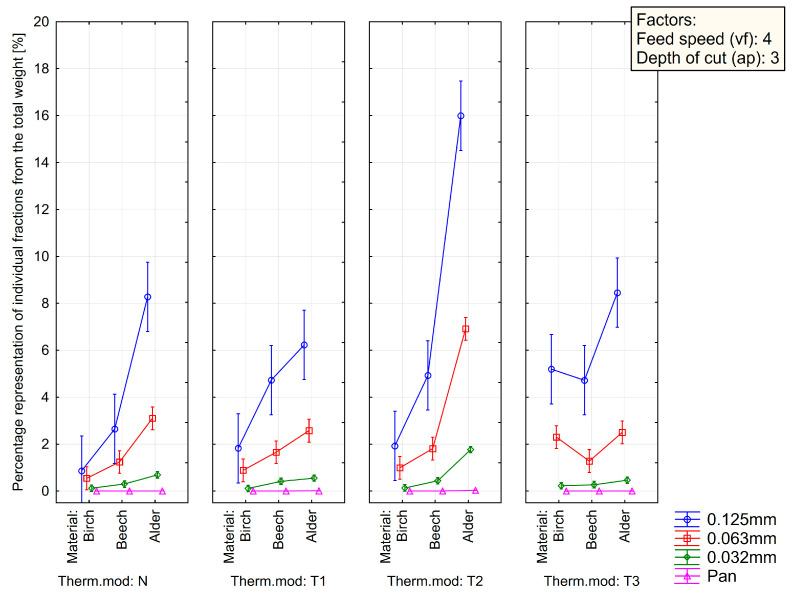
Sieve analysis of fine fraction obtained by the depth of cut by 3 mm and the feed rate of 4 m·min^−1^ (vf4-ap3).

**Figure 9:**
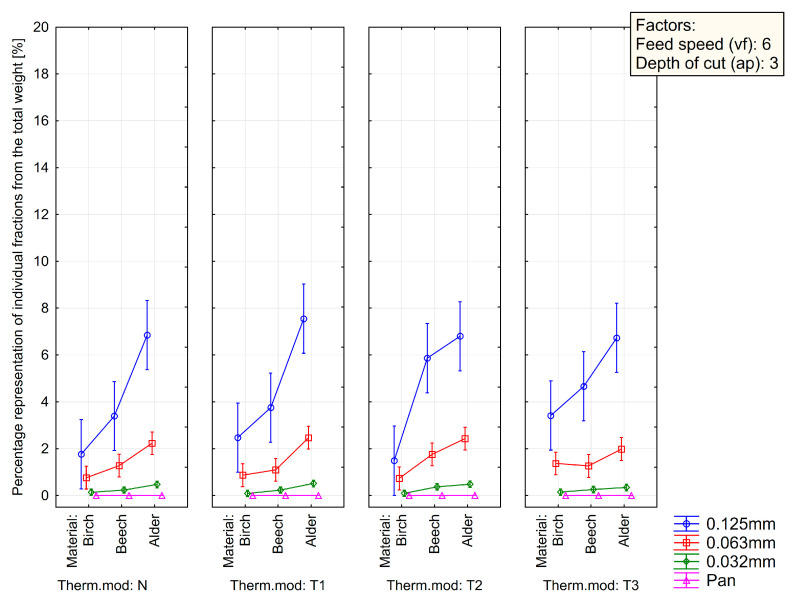
Sieve analysis of fine fraction obtained by the depth of cut by 3 mm and the feed rate of 6 m·min^−1^ (vf6-ap3).

Figure 7, Figure 8 and Figure 9 summarize the sieve analysis results of fine fraction obtained by the various feed rates.

Concerning the fine fractions formed, as already mentioned, no amount of dust fraction was observed in the sieve analysis in the range of 0.032 mm and less, as can be seen in Figure 7, Figure 8 and Figure 9. All these percentages represent the total weight share of the collected sample for each combination of material, thermal treatment, and feed rate. Comparing just the percentage content of fine dust particles by the type of wood species, it can be seen that alder wood created much more dust particles in the range of the so-called fine fractions. On the other hand, the birch created the lowest amount of fine dust particles. The lowest volume was observed in a natural state and in the thermal treatment of T1 and low feed speed of 2 m·min^−1^. Piernik et al. 2019 [49] suggest that milling of thermally treated pine wood does not cause significant differences in the overall grain distribution of dust, but it does affect the amount of ultrafine dust particles when using low cutting speeds, i.e., 1 m∙min^−1^. Taking into account another technological parameter, such is the depth of milling (they used 0.5 and 2.0 mm), they concluded that to reduce the amount of fine dust generated, the highest possible depth of machining should be used, which was taken into account in the methodology of this work using a depth of 3.0 mm [49].

The influence of the type of wood species, thermal treatment, and feed rate on the particle-size distribution of dust particles in the range of 0.125 mm to lower than 0.032 mm created during milling can be seen in Figure 3, Figure 4 and Figure 5. The content of the largest particles with a size greater than or equal to 2 mm is more than 95.63% for birch (T1 and feed speed by 2 m·min^−1^), 87.28% for beech (N and feed speed by 2 m·min^−1^), and 76.14% for alder (T1 and feed speed by 4 m·min^−1^). A maximal content of ultrafine particles that had been gathered on the sieves with a mesh size of 0.032 mm was 0.43% for birch (T2 and feed speed by 2 m·min^−1^), 1.15% for beech (T1 and feed speed by 2 m·min^−1^), and 2.53% for alder (T2 and feed speed by 4 m·min^−1^). The tested wood species vary in mechanical and physical properties.

Statistical analysis results for the 0.125 mm size fraction are, that the factors of the material were proven with statistical significance *p* < 0.000. The statistical importance of factor thermal treatment was also proven with *p* < 0.0159, but the feed rate was not proven with *p* < 0.0876. A combination of material and thermal treatment was proven with *p* < 0.259, but the other combinations were not proven to be statistically significant.

For the fractions of size 0.063 mm, the results are quite similar. Again, the statistical importance of the material was proven with the *p* < 0.000 and the same for the thermal treatment with the *p* < 0.0018. In this case, even the factor of feed rate was proven statistically significant with *p* < 0.000. The combination of material and thermal treatment was proven with *p* < 0.000, and the combination of material and feed rate with *p* < 0.0036. Thermal treatment and feed rate were not proven to be statistically significant with *p* < 0.14, but the combination of material, thermal modification, and feed rate was proven to be significant with *p* < 0.016. The same can be said about particles with a size of 0.032 mm. Again, the factor of material was proven with *p* < 0.000, a thermal treatment proven with *p* < 0.001, and a feed rate proven with *p* < 0.000. The combination of material and thermal treatment was again proven with *p* < 0.000, and the material and the feed rate were proven with *p* < 0.000. The combination of thermal treatment and feed rate was again not proven with *p* < 0.11, but the combination of material, thermal treatment and feed rate was proven with *p* < 0.04.

The most interesting part is the fractions with sizes smaller than 0.032 mm. Even when numbers gathered on the sieves, seem to be almost negligible, the statistical importance of the material was found and proven with *p* < 0.002. Furthermore, the combination of material and thermal treatment with *p* < 0.02 was proven, but all the other factors were not proven to be significantly important.

In general, it can be stated that:

- The level of statistical significance for material factor was proven in all four cases;
- The statistical importance of thermal treatment was not proven in all four cases;
- The statistical importance of feed rate was not proven in all four cases;
- And the statistical importance of combination material and thermal treatment was proven only for dust fractions lower than 0.032 mm.

The material itself and the combination of material and thermal treatment are significant in creating dust particles in the range of 0.032 mm and lower.

Based on the results obtained, it is not possible to conclude definitively whether thermal treatment affects the amount of dust formed, including fine fraction dust. The amount of milling dust formed from thermally treated wood under the three process temperature conditions shows no correlation. There is no correlation, which is more apparent for the feed speed. It would therefore be appropriate to carry out future studies on the influence of wood species on thermal treatment and, in a further step, on the processing, including the dust generated.

## 4. Conclusions

The study concerns an experimental comparative analysis of the effect of thermal treatment and feed rate on the fine dust fraction content created during the milling of three types of wood: birch, beech, and alder. As a result of heat treatment, there is a reduction in density for birch and alder, and for beech wood, there is no significant change in the material density. After dust granulometric analysis, particular emphasis was put on analyzing two dust fractions, the finest—smaller than 0.032 mm and the fine—in the range of 0.032 to 0.125 mm. The results analysis suggest that increasing the feed rate can reduce the content of fine dust particles in the range of 0.125–0.032 mm.

For birch- and beech wood, heat treatment increases the share of fine dust particles and for alder it decreases in the range of 0.125 mm and lower, compared to the natural state at a combination of the low feed rate of 2 m∙min^−1^.

The relation between feed rate and the wood type to the decreasing the content of dust particles in the range from 0.125 mm to 0.032 mm is still unclear. From a practical point of view, it cannot be clearly indicated that thermal treatment at such low temperatures affects the size of fine fractions, the differences between the variants are not large and may be due to the heterogeneous structure of the wood. The different density behavior during heat treatment in beech wood requires further investigation.

## Figures and Tables

**Table 1:** Physical properties and acronyms used for the thermal treatment variants used.

Type of Material	Density (*ρ*) (kg·m^−3^)	Thermal Treatment Mode
birch wood	649	N
	625	T1
	620	T2
	621	T3
beech wood	683	N
	670	T1
	682	T2
	684	T3
alder wood	523	N
	512	T1
	510	T2
	500	T3

**Table 2:** Modes of modification of blanks saturated with water vapor.

Modes	Saturated Steam Temperature (°C)			Pressure (N/mm^2^)	Duration of Steaming (Hours)		
	*t*_min_	*t*_max_	*t*_4_		*τ*_1_—Phase I	*τ*_2_—Phase II	Total Time
Mode T1	102.5	107.5	100	0.12	9.0	1.5	10.5
Mode T2	122.5	127.5	100	0.23			
Mode T3	132.5	137.5	100	0.31			

## Data Availability

The data presented in this study are available on request from the corresponding author.

## References

[1] Kauppinen T. (2000). Occupational Exposure to Carcinogens in the European Union. Occup. Environ. Med..

[2] Nasir V., Cool J. (2020). A Review on Wood Machining: Characterization, Optimization, and Monitoring of the Sawing Process. Wood Mater. Sci. Eng..

[3] Jacobsen G., Schaumburg I., Sigsgaard T., Schlünssen V. (2021). Wood Dust Exposure Levels and Respiratory Symptoms 6 Years Apart: An Observational Intervention Study Within the Danish Furniture Industry. Ann. Work. Expo. Health.

[4] Douwes J., Cheung K., Prezant B., Sharp M., Corbin M., McLean D., ‘t Mannetje A., Schlunssen V., Sigsgaard T., Kromhout H. (2017). Wood Dust in Joineries and Furniture Manufacturing: An Exposure Determinant and Intervention Study. Ann. Work. Expo. Health.

[5] Yuan Z., Khakzad N., Khan F., Amyotte P. (2015). Dust Explosions: A Threat to the Process Industries. Process Saf. Environ. Prot..

[6] Sydor M., Mirski R., Stuper-Szablewska K., Rogoziński T. (2021). Efficiency of Machine Sanding of Wood. Appl. Sci..

[7] Teuber L., Osburg V.-S., Toporowski W., Militz H., Krause A. (2016). Wood Polymer Composites and Their Contribution to Cascading Utilisation. J. Clean. Prod..

[8] Janiszewska D., Frackowiak I., Mytko K. (2016). Exploitation of Liquefied Wood Waste for Binding Recycled Wood Particleboards. Holzforschung.

[9] Demers P.A., Weinrich A.J. (2014). Wood Dusts. Encyclopedia of Toxicology.

[10] Top Y. (2020). Relationship between Employees’ Perception of Airborne Wood Dust and Ventilation Applications in Micro-Scale Enterprises Producing Furniture. Bioresources.

[11] Özkaya E., Aslan M.S.E. (2021). Occupational Allergic Contact Dermatitis: A 24-year, Retrospective Cohort Study from Turkey. Contact Dermat..

[12] Hancock D.G., Langley M.E., Chia K.L., Woodman R.J., Shanahan E.M. (2015). Wood Dust Exposure and Lung Cancer Risk: A Meta-Analysis. Occup. Environ. Med..

[13] Gu J., Kirsch I., Schripp T., Froning-Ponndorf F., Berthold D., Salthammer T. (2018). Human Exposure to Airborne Particles during Wood Processing. Atmos. Environ..

[14] Siew S.S., Martinsen J.I., Kjaerheim K., Sparén P., Tryggvadottir L., Weiderpass E., Pukkala E. (2017). Occupational Exposure to Wood Dust and Risk of Nasal and Nasopharyngeal Cancer: A Case-Control Study among Men in Four Nordic Countries-with an Emphasis on Nasal Adenocarcinoma. Int. J. Cancer.

[15] Lim S.S., Vos T., Flaxman A.D., Danaei G., Shibuya K., Adair-Rohani H., AlMazroa M.A., Amann M., Anderson H.R., Andrews K.G. (2012). A Comparative Risk Assessment of Burden of Disease and Injury Attributable to 67 Risk Factors and Risk Factor Clusters in 21 Regions, 1990–2010: A Systematic Analysis for the Global Burden of Disease Study 2010. Lancet.

[16] Directive 2004/37/EC of the European Parliament and of the Council of 29 April 2004 on the Protection of Workers from the Risks Related to Exposure to Carcinogens or Mutagens at Work (Sixth Individual Directive within the Meaning of Article 16(1) of Council Directive 89/391/EEC) (Codified Version)–European Sources Online. https://osha.europa.eu/en/legislation/directive/directive-200437ec-carcinogens-or-mutagens-work.

[17] Ding T., Zhao J., Zhu N., Wang C. (2020). A Comparative Study of Morphological Characteristics of Medium-Density Fiberboard Dust by Sieve and Image Analyses. J. Wood Sci..

[18] (1993). Workplace Atmospheres-Size Fraction Definitions for Measurement of Airborne Particles.

[19] (1995). Air Quality–Particle Size Fraction Definitions for Health-Related Sampling.

[20] Thomas R.J. (2013). Particle Size and Pathogenicity in the Respiratory Tract. Virulence.

[21] Micallef C.M., Shield K.D., Baldi I., Charbotel B., Fervers B., Ilg A.G.S., Guénel P., Olsson A., Rushton L., Hutchings S.J. (2018). Occupational Exposures and Cancer: A Review of Agents and Relative Risk Estimates. Occup. Environ. Med..

[22] Baran S., Teul I. (2007). Wood Dust: An Occupational Hazard Which Increases the Risk of Respiratory Disease. J. Physiol. Pharm..

[23] Proto A.R., Negri M., Marra M. (2009). Dust Exposures in the Wood Processing Industry in Northeast Italy. Proceedings of the International Sci-Entific Conference on Hardwood Processing.

[24] Hlásková L., Rogoziński T., Kopecký Z. (2016). Influence of Feed Speed on the Content of Fine Dust during Cutting of Two-Side-Laminated Particleboards. Drv. Ind..

[25] Pędzik M., Stuper-Szablewska K., Sydor M., Rogoziński T. (2020). Influence of Grit Size and Wood Species on the Granularity of Dust Particles during Sanding. Appl. Sci..

[26] Rogoziński T., Chuchala D., Pędzik M., Orlowski K.A., Dzurenda L., Muzinski T. (2021). Influence of Drying Mode and Feed per Tooth Rate on the Fine Dust Creation in Pine and Beech Sawing on a Mini Sash Gang Saw. Eur. J. Wood Wood Prod..

[27] Beljo-Lučić R., Čavlović A.O., Antonović A., Vujasinović E., Šimičić I. (2005). Properties of Chipped Wood Generated during Mechanical Wood Processing. Drv. Ind..

[28] Jehlička T., Sander J. (2019). Separation of Dust Particles in the Low-Pressure Pneumatic Conveying System. Agron. Res..

[29] Liang X., Yao Y., Xiao X., Liu X., Wang X., Li Y. (2022). Pressure-Steam Heat Treatment-Enhanced Anti-Mildew Property of Arc-Shaped Bamboo Sheets. Polymers.

[30] Ye C., Huang Y., Feng Q., Fei B. (2020). Effect of Hygrothermal Treatment on the Porous Structure and Nanomechanics of Moso Bamboo. Sci. Rep..

[31] Kolya H., Kang C.-W. (2021). Hygrothermal Treated Paulownia Hardwood Reveals Enhanced Sound Absorption Coefficient: An Effective and Facile Approach. Appl. Acoust..

[32] Kozakiewicz P., Laskowska A., Drożdżek M., Zawadzki J. (2022). Influence of Thermal Modification in Nitrogen Atmosphere on Physical and Technological Properties of European Wood Species with Different Structural Features. Coatings.

[33] Abe K., Yamamoto H. (2006). Change in Mechanical Interaction between Cellulose Microfibril and Matrix Substance in Wood Cell Wall Induced by Hygrothermal Treatment. J. Wood Sci..

[34] Tang T., Fei B., Song W., Su N., Sun F. (2022). Tung Oil Thermal Treatment Improves the Visual Effects of Moso Bamboo Materials. Polymers.

[35] Boonstra M.J., van Acker J., Tjeerdsma B.F., Kegel E.v. (2007). Strength Properties of Thermally Modified Softwoods and Its Relation to Polymeric Structural Wood Constituents. Ann. For. Sci..

[36] Yildiz S., Gezer E.D., Yildiz U.C. (2006). Mechanical and Chemical Behavior of Spruce Wood Modified by Heat. Build Environ..

[37] Mburu F., Dumarçay S., Bocquet J.F., Petrissans M., Gérardin P. (2008). Effect of Chemical Modifications Caused by Heat Treatment on Mechanical Properties of Grevillea Robusta Wood. Polym. Degrad. Stab..

[38] Bal B.C. (2014). Some Physical and Mechanical Properties of Thermally Modified Juvenile and Mature Black Pine Wood. Eur. J. Wood Wood Prod..

[39] Korkut S. (2012). Performance of Three Thermally Treated Tropical Wood Species Commonly Used in Turkey. Ind. Crops Prod..

[40] Garcia R.A., de Carvalho A.M., de Figueiredo Latorraca J.V., de Matos J.L.M., Santos W.A., de Medeiros Silva R.F. (2012). Nondestructive Evaluation of Heat-Treated Eucalyptus Grandis Hill Ex Maiden Wood Using Stress Wave Method. Wood Sci. Technol..

[41] Cademartori P.H.G., dos Santos P.S.B., Serrano L., Labidi J., Gatto D.A. (2013). Effect of Thermal Treatment on Physicochemical Properties of Gympie Messmate Wood. Ind. Crops Prod..

[42] Majka J., Sydor M., Pędzik M., Antov P., Krišťák Ľ., Kminiak R., Kučerka M., Rogoziński T. (2021). Quantifying the Finest Particles in Dust Fractions Created during the Sanding of Untreated and Thermally Modified Beech Wood. Bioresources.

[43] (2003). Moisture Content of a Piece of Sawn Timber-Part 1: Determination by Oven Dry Method.

[44] Dzurenda L. (2022). Mode for Hot Air Drying of Steamed Beech Blanks While Keeping the Colours Acquired in the Steaming Process. Acta Fac. Xylologiae.

[45] Kvietková M., Gašparík M., Gaff M. (2015). Effect of Thermal Treatment on Surface Quality of Beech Wood after Plane Milling. Bioresources.

[46] Yildiz S. (2002). Physical, Mechanical, Technological and Chemical Properties of Beech and Spruce Wood Treated by Heating. Ph.D. Thesis.

[47] Kminiak R., Kučerka M., Kristak L., Reh R., Antov P., Očkajová A., Rogoziński T., Pędzik M. (2021). Granulometric Characterization of Wood Dust Emission from Cnc Machining of Natural Wood and Medium Density Fiberboard. Forests.

[48] Kučerka M., Očkajová A. (2018). Thermowood and Granularity of Abrasive Wood Dust. Acta Fac. Xylologiae Zvolen.

[49] Piernik M., Rogoziński T., Krauss A., Pinkowski G. (2019). The Influence of the Thermal Modification of Pine (*Pinus sylvestris* L.) Wood on the Creation of Fine Dust Particles in Plane Milling. J. Occup. Health.

